# Two C18 hydroxy-cyclohexenone fatty acids from mammalian epidermis: Potential relation to 12*R*-lipoxygenase and covalent binding of ceramides

**DOI:** 10.1016/j.jbc.2023.104739

**Published:** 2023-04-21

**Authors:** Alan R. Brash, Saori Noguchi, William E. Boeglin, M. Wade Calcutt, Donald F. Stec, Claus Schneider, Jason M. Meyer

**Affiliations:** 1 Department of Pharmacology, Vanderbilt University School of Medicine, Nashville, Tennessee, USA; 2 Department of Biochemistry, Vanderbilt University School of Medicine, Nashville, Tennessee, USA; 3 Department of Chemistry, Vanderbilt University, Nashville, Tennessee, USA; 4 Department of Dermatology, Vanderbilt University Medical Center, and Dermatology Service, Department of Veterans Affairs, Tennessee Valley Healthcare System, Nashville, Tennessee, USA

**Keywords:** ceramide, corneocyte, epidermis, essential fatty acid, linoleic acid, lipoxygenase, mass spectrometry, NMR

## Abstract

A key requirement in forming the water permeability barrier in the mammalian epidermis is the oxidation of linoleate esterified in a skin-specific acylceramide by the sequential actions of 12*R*-lipoxygenase, epidermal lipoxygenase-3, and the epoxyalcohol dehydrogenase SDR9C7 (short-chain dehydrogenase-reductase family 7 member 9). By mechanisms that remain unclear, this oxidation pathway promotes the covalent binding of ceramides to protein, forming a critical structure of the epidermal barrier, the corneocyte lipid envelope. Here, we detected, in porcine, mouse, and human epidermis, two novel fatty acid derivatives formed by KOH treatment from precursors covalently bound to protein: a “polar” lipid chromatographing on normal-phase HPLC just before omega-hydroxy ceramide and a “less polar” lipid nearer the solvent front. Approximately 100 μg of the novel lipids were isolated from porcine epidermis, and the structures were established by UV-spectroscopy, LC–MS, GC–MS, and NMR. Each is a C18 fatty acid and hydroxy-cyclohexenone with the ring on carbons C_9_–C_14_ in the polar lipid and C_8_–C_13_ in the less polar lipid. Overnight culture of [^14^C]linoleic acid with whole mouse skin *ex vivo* led to recovery of the ^14^C-labeled hydroxy-cyclohexenones. We deduce they are formed from covalently bound precursors during the KOH treatment used to release esterified lipids. KOH-induced intramolecular aldol reactions from a common precursor can account for their formation. Discovery of these hydroxy-cyclohexenones presents an opportunity for a reverse pathway analysis, namely to work back from these structures to identify their covalently bound precursors and relationship to the linoleate oxidation pathway.

Life on dry land requires the presence of a barrier to water loss to prevent dehydration, and this is provided by the outermost layer of the epidermis, the stratum corneum (1). Lipids are important structural components of the barrier and consist of “a roughly equimolar mixture of ceramides (45–50% by weight), cholesterol (25%), and free fatty acids (10–15%) plus less than 5% each of several other lipids” (1). These lipids mainly serve to fuse together the terminally differentiated anucleated corneocytes that now have the plasma membrane replaced by a polymerized protein coat known as the corneocyte envelope (CE) (2, 3). Crucially, there is a monomolecular layer of lipid covalently bonded to the outside surface of the CE of each corneocyte, forming a substructure known as the corneocyte lipid envelope (CLE) (reviewed in Refs. (4, 5, 6)). The molecular composition of the covalently bonded lipids of the CLE was determined in the late 1980s onward and shown to account for about 10% of the barrier ceramides along with covalently bound fatty acids and omega-hydroxy fatty acids (7, 8, 9, 10). The CLE is considered to function as a scaffold for the majority of the remaining lipid, which is fused between the corneocytes in well-organized layers of intercellular free lipids visible by EM as layers of lipid lamellae (*e.g.*, (1, 4, 5, 6)).

One of the connections to lipids and the epidermal permeability barrier dates back to the discovery of essential fatty acids (EFA) and the fact that barrier function is compromised by EFA deficiency resulting in transepidermal water loss and development of a hyperproliferative scaly skin (11, 12). Linoleic acid (9*cis*,12*cis*-C18:2) is the one and only EFA with an obligatory role in skin barrier formation (13, 14, 15, 16, 17). As EFA deficiency develops in a rodent model, the resulting transepidermal water loss is associated with around a 50% decrease in covalently bound ceramides (18). Current knowledge places linoleate metabolism through an oxidative pathway initiated by 12*R*-lipoxygenase (12*R*-LOX) as key to its functional role in barrier formation (19, 20, 21, 22). Genetic deficiency in 12*R*-LOX or of the subsequent enzymes in the pathway (eLOX3 [epidermal lipoxygenase-3] and SDR9C7 [short-chain dehydrogenase-reductase family 7 member 9]) results in severe skin barrier disruption, which in common with deficiencies in other barrier genes is neonatal lethal in mice due to uncontrollable transepidermal water loss (22, 23, 24, 25) and causes congenital ichthyosis (scaly skin disease) in humans. Remarkably, the structural defect associated with these 12*R*-LOX pathway gene deficiencies is an almost complete lack of covalently bound ceramides in the epidermal barrier and the absence of the CLE (22, 25, 26, 27).

There are two further issues of background that should be helpful to have briefly introduced. The first is the nature of the substrate and transformations by the enzymes 12*R*-LOX, eLOX3, and SDR9C7 in epidermal barrier formation. The substrate is the skin-specific acylceramide, Cer-EOS (Ceramide-Esterified Omega-hydroxy Sphingosine), that has mainly linoleate as the fatty acid esterified to the ω-hydroxyl of its amide-linked long-chain fatty acid (Fig. 1 (26)). The three enzymes act in series on Cer-EOS to produce Cer-EOS-epoxy-ketone, an oxidized derivative with the linoleate ester converted to 9*R*,10*R*-*trans*-epoxy-11*E*-13-keto-octadecenoate (22). The second issue relates to the knowns and unknowns regarding the nature of the covalent binding to protein. It is firmly established that at least 30% of the binding is ester linkage of the ω-hydroxyl of ceramide omega-hydroxy sphingosine (Cer-OS) to glutamate residues in the protein (9) (Fig. 1). Another possibility, proposed though not established, is direct coupling between Cer-EOS-epoxy-ketone and protein *via* the chemical reactivity of the epoxy-ketone moiety itself (22). Whatever is happening, the oxidations through the 12*R*-LOX pathway are required to initiate essentially all the covalent binding (22, 25, 26, 27). To the best of our knowledge, only one study has examined directly the protein–lipid linkages, the one already referenced to ceramide binding (9). Most commonly, covalent binding is analyzed by removal of free lipids by extensive washing of epidermis with MeOH/CHCl_3_ followed by mild alkaline hydrolysis to release lipids ester bonded to protein. Using this approach, herein we describe the identification of novel components of the covalently bound lipids of the skin permeability barrier and their potential connection to 12*R*-LOX metabolism.

**Figure 1:**
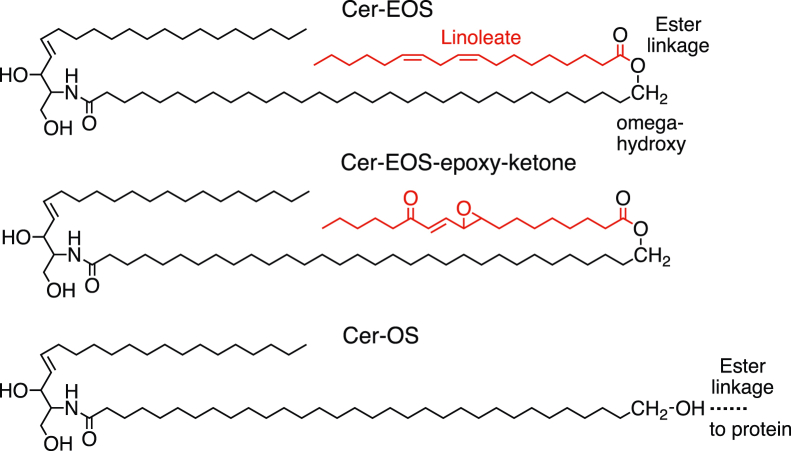
**Structures of the skin-specific ceramides Cer-EOS, Cer-EOS-epoxy-ketone, and Cer-OS.** Cer-EOS is converted to Cer-EOS-epoxy-ketone by the consecutive actions of 12*R*-LOX, eLOX3, and SDR9C7 required for covalent binding of ceramides to protein and formation of the CLE (main text). The coupling of Cer-EOS-epoxy-ketone to protein is predicted from the chemical reactivity of the epoxy-ketone moiety, although not so far demonstrated. The covalent binding of epidermal ceramides to protein is known to involve, in part, ester linkage of Cer-OS to glutamate residues (9). Cer-EOS, Ceramide-Esterified Omega-hydroxy Sphingosine; Cer-OS, ceramide omega-hydroxy sphingosine; CLE, corneocyte lipid envelope; eLOX3, epidermal lipoxygenase-3; 12*R*-LOX, 12*R*-lipoxygenase; SDR9C7, short-chain dehydrogenase-reductase family 7 member 9.

## Results

### Two novel lipids detected after alkaline hydrolysis of epidermal proteins

**Figure 2:**
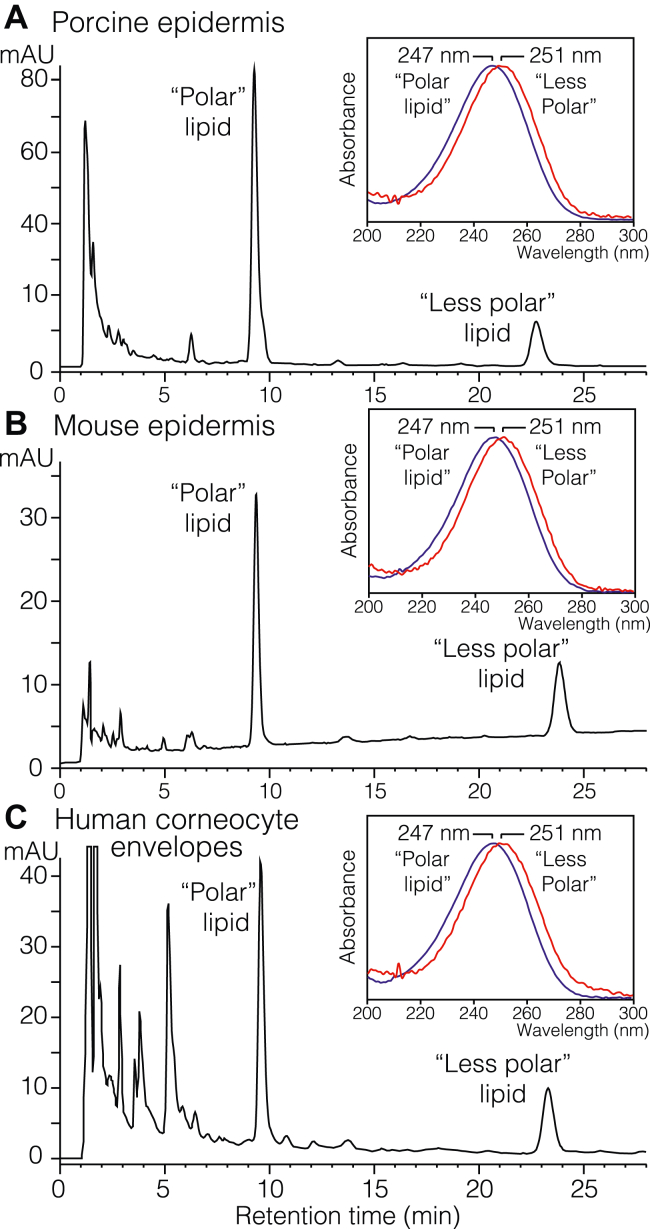
**Reverse-phase HPLC (RP-HPLC) analysis of lipids covalently bound to epidermal protein.** Covalently bound lipids were prepared as described in the main text and analyzed using a Waters Symmetry 5 μ C18 column (15 × 0.21 cm) with an isocratic solvent of acetonitrile/water/glacial acetic acid (40:60:0.01 by volume) at a flow rate of 0.3 ml/min and illustrating the UV recording at 235 nm. *A*, porcine epidermal lipids recovered from covalent binding to protein. *B*, mouse pup epidermal lipids recovered from covalent binding to protein. *C*, human epidermal corneocyte envelope lipids recovered from covalent binding to protein. The *insets* show the UV spectra of the “polar” and “less polar” lipids in each chromatogram with their λmax of 247 nm (polar lipid) and 251 nm (less polar lipid) in this CH_3_CN:H_2_O (40:60, v/v) solvent.

Epidermal tissue is extracted multiple times with MeOH/CHCl_3_ to remove free lipids, and then lipids ester bound to protein are released by alkaline hydrolysis using 1 M KOH in 95% methanol overnight at room temperature. Water is then added, the suspension is neutralized and slightly acidified to pH 4 to pH 5, and the released lipids recovered by Bligh and Dyer extraction (28). Analysis of pig and mouse epidermis, and human isolated CEs by reversed phase (RP)-HPLC with UV detection showed the elution of two unidentified products with prominent absorbance in the 235 nm channel and designated as the “polar” lipid and the later eluting “less polar” lipid (Fig. 2). They have very similar UV spectra, each with a smooth symmetrical chromophore characteristic of a conjugated enone and with a relatively high λmax indicative of the double bond being in a ring (Fig. 2, *insets*) (29). In the RP-HPLC column solvent (CH_3_CN/H_2_O/glacial acetic acid [HAc] 40:60:0.01), the λmax values are 247 nm (polar lipid) and 251 nm (less polar) and 245 and 248 nm, respectively, in methanol. Fig. S1 illustrates the corresponding separation by straight-phase (SP) HPLC-UV.

### Assignment of a molecular formula

Reversed-phase LC with high-resolution mass spectrometry (MS) electrospray in both positive and negative ions gave only negative ion signals and the base peak for polar and less polar lipids at *m/z* 309.2078 and 309.2079, respectively, defining their identical molecular composition of C_18_H_29_O_4_ (calculated 309.2071). A few seconds treatment with diazomethane extended their retention times on RP-HPLC as expected for a methyl ester derivative, and subsequent GC–MS analyses confirmed that both polar and less polar lipids are C18 fatty acids each with one double bond in a ring, one ketone, and one hydroxyl (C_18_H_30_O_4_). An extensive series of derivatives of each compound were analyzed by GC–MS, to be considered further. Initially, it is instructive to review the proton NMR analyses.

### Proton NMR analysis of the two products

**Figure 3:**
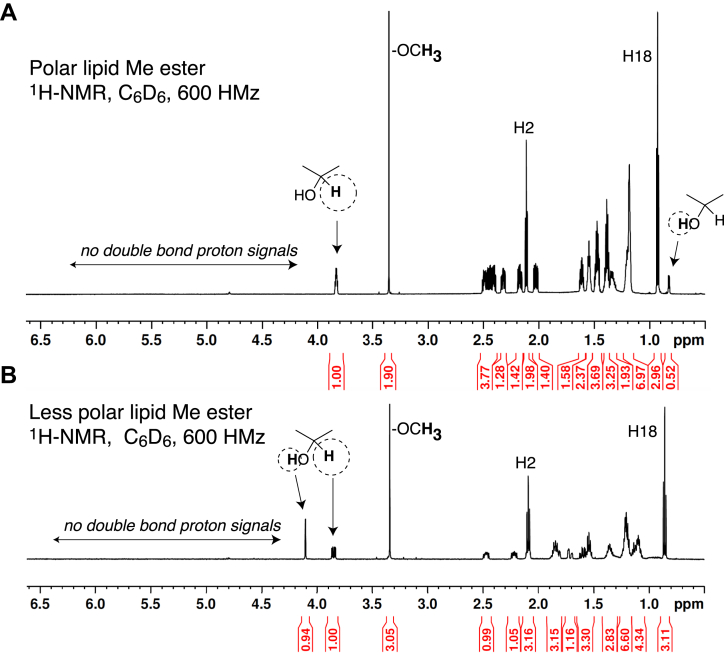
**Proton NMR spectrum of the polar and less polar lipids as the methyl ester.***A*, polar lipid methyl ester. *B*, less polar lipid methyl ester. Both spectra were recorded in d_6_-benzene at 600 MHz. Comparative peak areas are given in the *red text* below each 1D spectrum. The most distinctive features are labeled in the figure. The downfield chemical shift of the HO- proton in the less polar product (signal at 4.1 ppm) compared with the polar product (signal at 0.8 ppm) is due to hydrogen bonding in the α-ketol of the less polar molecule. Detailed analysis of the assignments and coupling are given in Figures 4 and 5 and supporting information (Figs. S2–S9).

**Figure 4:**
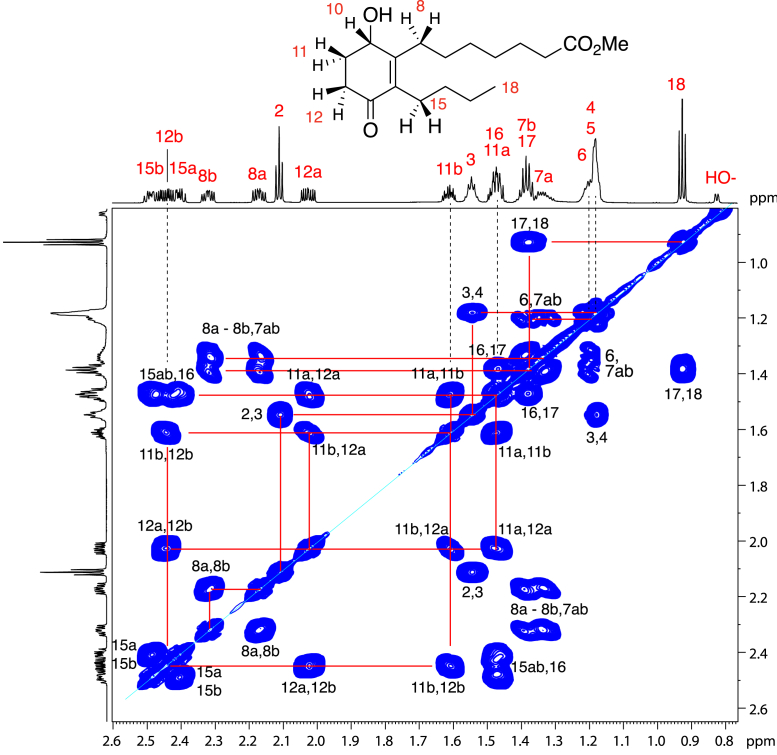
**Partial**^**1**^**H-NMR spectrum (800 MHz, 0.7–2.6 ppm) and COSY analysis of the polar lipid methyl ester in d**_**6**_**-benzene**.

**Figure 5:**
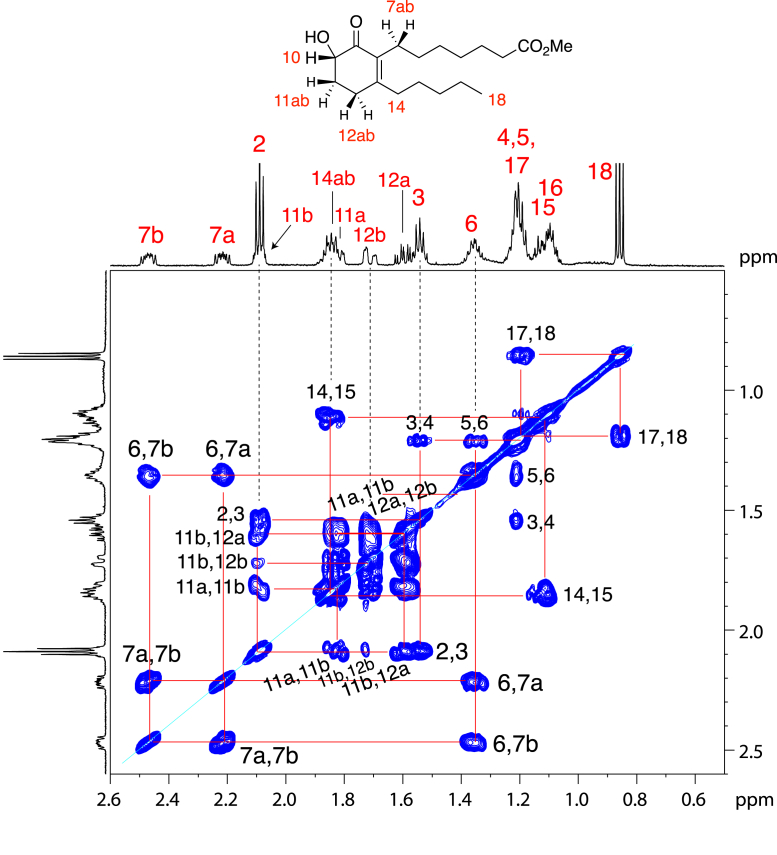
**Partial**^**1**^**H-NMR spectrum (600 MHz, 0.5–2.6 ppm) and COSY analysis of the less polar lipid methyl ester in d**_**6**_**-benzene**.

NMR-compatible quantities of each product were recovered from 20 × 20 cm sections of porcine epidermis, providing after purification approximately 100 μg of each, based on quantitation by UV and assuming a molar extinction coefficient of 12,500 M^−1^ cm^−1^ (within the range of 10,000–15,000 typical for a conjugated enone (29)). For the polar lipid, proton NMR spectra were recorded mainly as the methyl ester derivative in d_6_-benzene along with COSY, heteronuclear single quantum coherence (HSQC), and heteronuclear multiple bond correlation (HMBC) at both 600 and 800 MHz and a NOESY spectrum at 800 MHz (Figs. S2–S7). The methyl ester of the less polar lipid was recorded in d_6_-benzene with COSY, HSQC, and HMBC (Supporting information, Figs. S8 and S9). The full ^1^H-NMR spectra are shown in Figure 3. Strikingly, there are no signals above ∼4 ppm in either spectrum, indicating the absence of a proton or protons on a double bond (signals expected at ∼5–6 ppm). This indicates that in each product the double bond (known to exist from the UV spectrum) is tetrasubstituted with carbons. This is also consistent with the double bond being in a ring (as suggested by the high λmax of the UV spectra) and with two carbon side chains. The signal at ∼3.8 ppm in each spectrum is the geminal proton on the carbon with a hydroxyl group, and H2 and H18 are the other obvious assignments. All proton signals can be assigned to carbons based on the COSY spectra and supported by HSQC and HMBC. A close-up view of the 0.5 to 2.5 ppm regions of the COSY analyses illustrates the strong signals and overall quality of these data (Figs. 4 and 5).

### GC–MS analyses

Multiple derivatives analyzed in the electron impact (EI) mode fully supported the molecular composition of both products, and key spectra, taken in combination with the UV spectra and ^1^H-NMR data, provided critical information that helped secure their molecular structures. All told, EI mass spectra were acquired on the methyl ester TMS ether and TMS ester TMS ether derivatives, including after derivatization of the ketone to the methoxime or d3-methoxime, also after NaBH_4_ reduction of the ketone, and after catalytic hydrogenation of the double bond.

**Figure 6:**
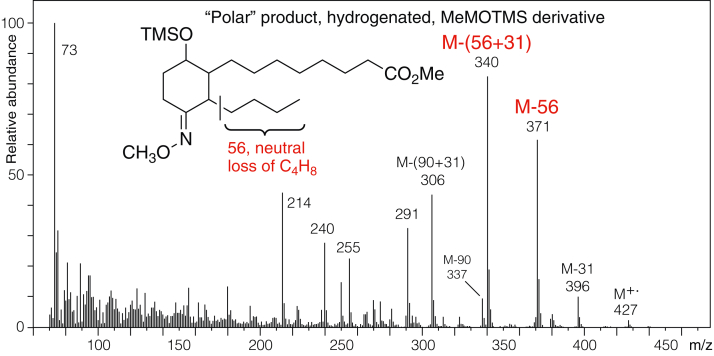
**Electron impact (EI) mass spectrum of the hydrogenated polar lipid as the methyl ester methoxime TMS ether derivative**.

For the polar lipid, the spectrum of the hydrogenated molecule as the methyl ester, methoxime, TMS ether is informative (Fig. 6). The molecular ion at *m/z* 427 and the M-31 ion at *m/z* 396 confirm the expected molecular mass. Structurally diagnostic are the prominent ions at *m/z* 371 (neutral loss of 56, C_4_H_8_) and *m/z* 340 (loss of 31 + 56). The neutral loss of 56 can only be accounted for by McLafferty rearrangement from the C13 methoxime (30), thus defining the lower side chain of the polar lipid as C_4_H_9_. From this and the NMR connectivities, only a six-membered ring can rationalize the remainder of the structure. Placing the parent ketone at C13 in a six-membered ring, together with the NMR data, allows definition of the structure of the polar lipid as the 10-hydroxy-13-keto-cyclohexenone illustrated in the figures.

**Figure 7:**
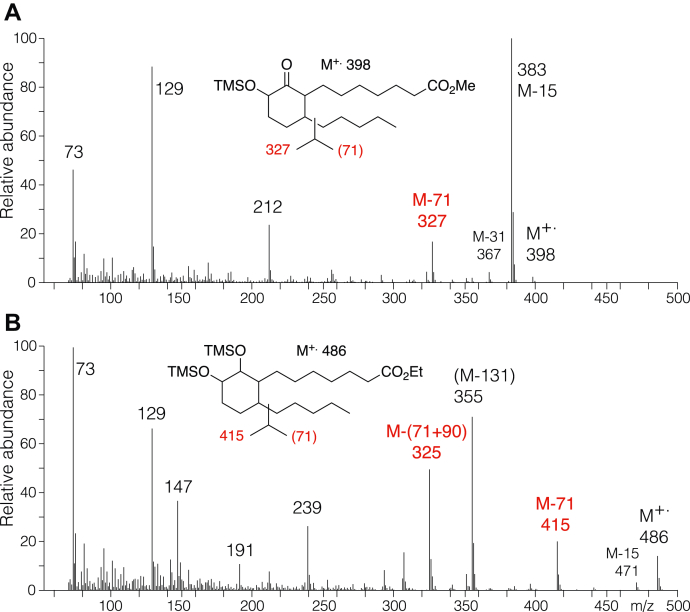
**Electron impact (EI) mass spectrum of the hydrogenated less polar lipid.** The (*A*) methyl ester TMS ether and (*B*) NaBH_4_-reduced ethyl ester TMS ether derivatives.

For the less polar lipid, the EI mass spectra of several derivatives of the hydrogenated molecule gave diagnostic M-71 ions. This is exemplified in the spectra of the hydrogenated less polar lipid as the methyl ester TMS ether derivative or the NaBH_4_-reduced ethyl ester TMS ether (Fig. 7). Both spectra display strong M-71 ions, indicative of loss of a pentyl side chain. Taken together with the NMR data showing the connections between the H10 geminal hydroxyl proton and coupling through H11ab to H12ab, the results from GC–MS help establish the structure of the less polar lipid as a 9-keto-10-hydroxy-cyclohexenone ring with side chains emanating from carbons 8 and 13.

### Stereochemical analysis

**Figure 8:**
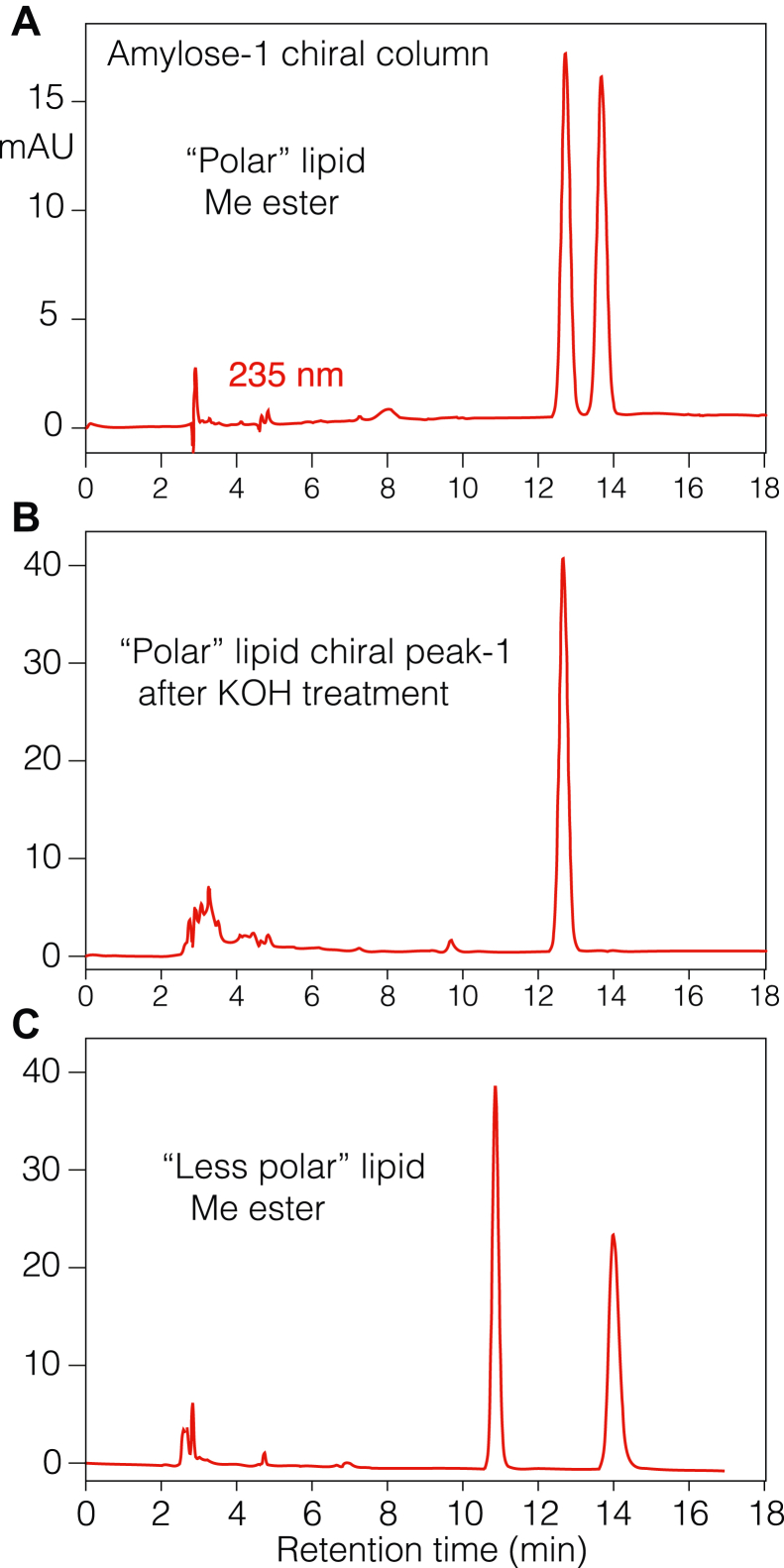
**Chiral HPLC of the polar and less polar lipids.***A*, “polar” lipid methyl ester analyzed using an Amylose-1 chiral column (Phenomenex, 3 μm, 25 × 0.46 cm), a solvent of hexane/MeOH/EtOH/glacial acetic acid (HAc) (100:5:5:0.02 by volume), and a flow rate of 1 ml/min with UV detection at 235 nm. *B*, “polar” lipid chiral peak-1 from another injection was collected, treated with 1 M KOH in 95% MeOH, extracted, remethylated, purified by SP-HPLC, and then reanalyzed as above. *C*, “less polar” lipid methyl ester analyzed under the same conditions.

Both products were analyzed by chiral column HPLC and shown to be racemic (Fig. 8). To check whether the alkaline hydrolysis conditions used to recover these lipids from the epidermis were responsible for their racemization, the first-eluting enantiomer of the polar lipid was collected and subjected to overnight treatment with 1 M KOH in 95% MeOH. After extraction, remethylation with diazomethane and repurification by SP-HPLC, analysis on the chiral column showed the polar lipid methyl ester remained a pure enantiomer (Fig. 8*B*).

### Transformations of [^14^C]linoleic acid in mouse skin *ex vivo*

**Figure 9:**
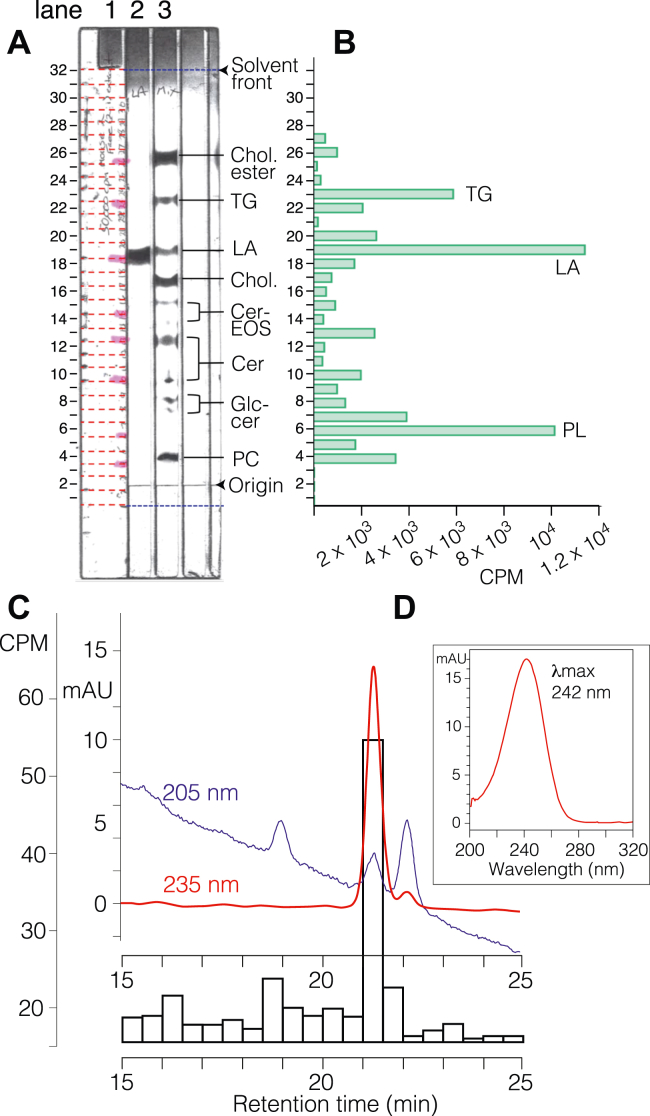
**TLC of the free lipids and straight-phase (SP-HPLC of the polar lipid after incubation of [**^**14**^**C]linoleic acid with mouse skin *ex vivo.*** [^14^C]Linoleic acid was applied topically to whole mouse skin *ex vivo* and incubated overnight. *A*, TLC separation of free lipids extracted from the epidermis (details in the Experimental procedures section). Lane 1 ran the radioactive-free lipid extract from epidermis, lane 2 unlabeled authentic linoleic acid, and lane 3 a selection of unlabeled lipid standards. After development, lane 1 was split into 32 zones, which were scraped into vials for liquid scintillation counting. The plate was then sprayed for detection of the cold standards in lanes 2 and 3 producing the bands shown. *B*, ^14^C profile of free lipids from lane 1 of the TLC plate. *C*, after extraction of the free lipids and alkaline hydrolysis of the epidermal proteins, a radiolabeled peak of “polar” lipid was collected on reverse-phase (RP)-HPLC and further purified by SP-HPLC with the profiles shown above at 205 nm (*blue*) and 235 nm (*red*). Column: Thomson 5 μm silica (25 × 0.46 cm), solvent hexane/IPA (isopropanol)/glacial acetic acid (HAc) (90:10:0.02 by volume) with a flow rate of 0.5 ml/min. Fractions were collected every 30 s and counted for at least 40 min each. *D*, UV spectrum of the radiolabeled peak, λmax 242 nm in SP-HPLC solvent, a match for the polar product. Cer, ceramide; Chol., cholesterol; LA linoleic acid; PC, phosphatidylcholine; PL, phospholipid; TG, triglyceride.

To probe for a potential connection between the two oxidized C18 fatty acids and the metabolism of linoleic acid in the epidermis, we topically applied [^14^C]linoleic acid onto the outside surface of intact mouse pup skin *ex vivo* and incubated overnight at 37 °C. Next day, the whole skin was treated with dispase (Roche) to allow recovery of the epidermis, which was then homogenized and extracted extensively with CHCl_3_/MeOH to remove free lipids. TLC analysis of the free lipids in comparison to a selection of authentic standards indicated that the linoleic acid had been extensively transformed during the overnight incubation with whole skin (Figs. 9, *A* and *B* and S10); the main bands probably corresponding to triglyceride, unmetabolized linoleic acid, possibly Cer-EOS in the middle of the plate and phospholipids at lower Rf values. The well-extracted protein pellet was subsequently treated with 1 M KOH in 95% MeOH to release covalently bound lipids and the Bligh and Dyer extract analyzed initially by RP-HPLC. A small radioactive peak at the expected retention time of the polar lipid showed its characteristic UV spectrum. This peak was further resolved by SP-HPLC (Fig. 9*C*), again showing the characteristic UV spectrum of the polar lipid (Fig. 9*D*). Counting of 0.25 ml fractions showed a peak of radioactivity coinciding with elution of the polar lipid (Fig. 9*C*). An estimate of the specific activity of the radioactive peak indicated in the order of 10- to 20-fold dilution of the linoleic acid applied to the whole skin. From another experiment, we identified ^14^C radiolabel in the less polar product (Fig. S11).

## Discussion

### Covalently bound lipids in the epidermal barrier

The constituents of the covalent lipid binding were identified in the late 1980s mainly by TLC and gas–liquid chromatography and assigned as ω-hydroxy-ceramides (Cer-OS and Cer-OH, 78.1%), ω-hydroxy-ultra–long-chain fatty acids (C30–C34, 9.4%), and free fatty acids (12.7%) (7) and similarly confirmed a few years later (8). Our approximations of the abundance of the polar and less polar lipids are in different units (50–100 μg/20^2^ cm epidermis) and not comparable to the earlier estimates. Nonetheless, they appear to be relatively abundant and probably contributed in the earlier work to the TLC profile around the area of the ω-hydroxy-fatty acids. Furthermore, they are recovered from the epidermis of three mammalian species, and in the case of the human samples, from CEs prepared from isolated stratum corneum, indicating their origin from components of the epidermal barrier.

### Novel structures

The six-membered carbon ring of the two fatty acids recovered from covalent binding in the epidermis is, to the best of our knowledge, unprecedented among naturally occurring eicosanoids or other oxylipin fatty acids. Although both products are racemic, they were recovered in NMR-compatible quantities, indicating a comparative abundance and suggestive of the involvement of enzymatic production. Furthermore, they appeared as two structures on HPLC and not the complex mix of isomers that might be expected *via* an autoxidation mechanism.

### An interesting dilemma in the structural analysis of the two lipids

**Figure 10:**
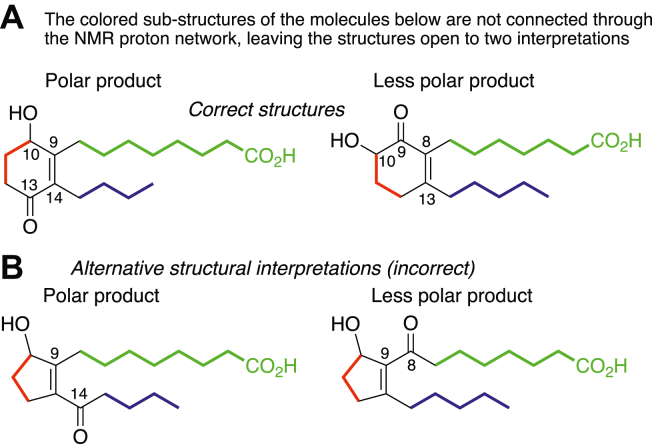
**Proton NMR of the two lipids has proton–proton connections in three separate sections of the molecules.***A*, correct structures with the six-membered ring; *B*, incorrect structures. The double bond and ketone split the proton connectivities into three sections, allowing two structural interpretations of the proton NMR with a five- or six-membered ring. GC–MS (Figs. 6 and 7) and the proposed mechanism of formation are only compatible with the six-membered ring.

The analytical data have a second possible interpretation, and although we reject this second possibility on a number of grounds, the analyst should find the issues of interest. Originally, we had considered that the two lipids might have a five-membered ring containing the hydroxyl, with the ketone on an adjacent side chain. These alternative structures (Fig. 10) have the identical molecular composition, a similar UV spectrum is predicted, and some of the GC–MS mass spectra are supportive or at least compatible. The structural ambiguity results from the tetrasubstituted double bond and the ketone splitting the observable proton NMR connections into three separate sections, unconnected from each other (Fig. 10). With no close homologs of defined structure to establish NMR ppm values, based on these data alone, a level of ambiguity has to be solved by other approaches.

Incidentally, the NMR proton connections throughout the structure of these two lipids would be completed after hydrogenation of the double bond, allowing a complete “read” around the carbon ring and out to the side chains. However, aside from any losses expected in the analytical manipulations, hydrogenation creates four diastereomers at the junction of the carbon ring and side chains (*cis,cis*, *cis,trans*, *trans,trans*, and *trans,cis*), which greatly reduces the yield after purification of a single entity for NMR analysis. Currently, we have insufficient product to take this approach and obtain sufficiently strong and definitive NMR connectivities.

The compelling evidence establishing a cyclohexenone ring rests on the combination of UV, NMR, and EI mass spectra. As noted in the Results section, the EI mass spectra in Figures 6 and 7 define the omega chain of each of the two products and are only consistent with the six-membered ring. In the case of the polar lipid, the mass spectrum of the hydrogenated molecule as the methyl ester methoxime derivative shows the prominent loss of 56 amu (C_4_H_8_), readily explained by McLafferty rearrangement, which entails chain cleavage beta to a ketone or a nitrogen substituent and neutral loss of the side chain (30); in the case of the alternative five-membered ring with exocyclic ketone at C14, the equivalent rearrangement would lead instead to loss of C_3_H_6_ (42 amu), not observed. For the less polar lipid, the loss of 71 amu (Fig. 6 and other derivatives) defines the omega side chain and the location of the cyclohexanone ring. This and related GC–MS data together with the NMR data leave only one secure interpretation of the two structures as hydroxy-cyclohexenones.

### A proposed mechanistic origin of the two lipids

**Figure 11:**
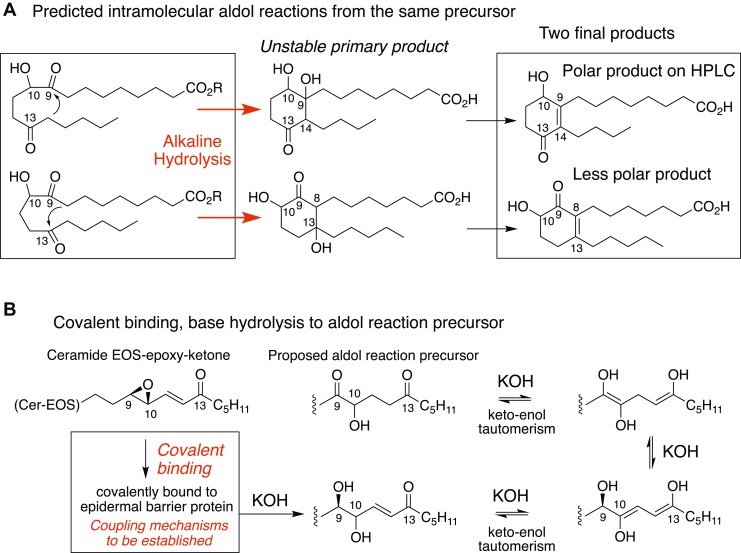
**Proposed formation of the polar and less polar lipids from a common precursor *via* intramolecular aldol reactions.***A*, predicted mechanism of formation of the polar and less polar lipids *via* intramolecular aldol reactions from a common precursor. The KOH treatment used to release lipids ester bound to epidermal proteins provides the conditions for the aldol condensations that can account for the six-membered rings, the locations of the ketone and hydroxyl groups, and the differing side-chain/ring junctions at carbons 9 and 14 in the polar lipid (*top*) and 8 and 13 in the less polar lipid (*below*). *B*, a proposed pathway whereby chiral and covalently bound C18 fatty acids oxidized on carbons 9, 10, and 13 are transformed *via* KOH-induced ester hydrolysis and ketoenol tautomerisms to the racemic 9,13-diketo-10-hydroxy aldol reaction precursor. The precise structures of the covalently bound lipids remain to be determined.

Any remaining questions related to the structures of the two lipids are well addressed by the proposed mechanism of formation that accounts for the two unusual hydroxy-cyclohexenones with their different-length side chains. The two lipids are recovered by overnight alkaline hydrolysis of epidermal proteins with 1 M KOH in 95% MeOH. This alkali treatment provides the conditions for intramolecular aldol reactions giving two unstable cyclic intermediates that readily dehydrate to form the hydroxy-cyclohexenones of the polar and less polar lipids (Fig. 11). In the proposed 1,5-diketo precursor, deprotonation α to one of the ketones provides the nucleophile to attack the C=O carbon on the other ketone, forming, after dehydration of the initial unstable product, a cyclohexenone (in fact a hydroxy-cyclohexenone because of the hydroxyl originally present). In the proposed precursor, the ketone at C9 is the more electrophilic, suggesting that formation of the polar product (Fig. 11, *top pathway*) is the more preferred. We were successful in isolating more of the polar product, which may be a reflection of this mechanistic preference. The chemistry of intramolecular aldol condensation is described in the two steps shown with the second step of dehydration of the intermediate facilitated by “heat,” which we did not intentionally apply, perhaps limiting the final yields.

### Potential connection to the 12*R*-LOX pathway and barrier formation

Currently, the final known product of the 12*R*-LOX pathway is the 9*R*,10*R*-*trans*-epoxy-11*E*-13-keto-octadecenoate esterified in Cer-EOS (Figs. 1*B* and 11), and formation of this Cer-EOS-epoxy-ketone is required for covalent binding and formation of the CLE (22). The proposed 9,13-diketo-10-hydroxy precursor of the polar and less polar lipids shares with the epoxy-ketone the same oxidizable positions of linoleic acid (C_9_–C_13_). And, importantly, linoleate is the only available C18:2 substrate in the epidermal barrier (13, 14, 15, 16, 17). Formation of the aldol reaction precursor is attributable to the base hydrolysis conditions used to recover the lipids covalently bound to protein (Fig. 11*B*). As alkaline hydrolysis releases the protein-bound esterified lipids, it will promote keto-enol tautomerisms that can account for production of the 9,13-diketo-10-hydroxyl (aldol reaction precursor) (Fig. 11*B*) and for its further conversion to the polar and less polar lipids (Fig. 11*A*). To further test for the connection between the linoleate oxidation pathway and the polar and less polar products, we tested for the potential transformation of [1-^14^C]linoleic acid to the ^14^C-labeled polar and less polar products after its topical application to intact mouse pup skin *ex vivo*. After overnight incubation, TLC analysis of the freely extractable lipids revealed the remarkable extent of incorporation of the [^14^C]linoleic acid to lipids with the polarity of triglycerides, ceramides (*cf*., studies in the rat (31)), and phospholipids (Fig. 9, *A* and *B*). Even more remarkably, after removal of the free lipids and the usual alkaline hydrolysis of the proteins, we detected radiolabeled polar and less polar products, identified from the retention times on RP-HPLC and SP-HPLC and the characteristic UV spectra (Figs. 9, *C* and *D* and S11).

Altogether, the evidence as it stands suggests that in normal epidermis there is a substantial quantity of covalently bound precursor of the polar and less polar lipids, with the strong implication that this is produced *via* the established 12*R*-LOX oxidative pathway. Earlier, we had detected traces of intact Cer-EOS-epoxy-ketone recoverable from thoroughly extracted epidermal protein and attributed this to reversible adduct formation (26). Here, we have evidence of binding on a more extensive scale, reinforcing the proposal that there is significant covalent binding of the 12*R*-LOX pathway products. One avenue to exploring how the 12*R*-LOX pathway links to its required role in covalent binding and formation of the CLE is to understand the immediate fate of its product, the Cer-EOS-epoxy-ketone, an approach that equates to a forward pathway analysis. The discovery of these unusual hydroxy-cyclohexenone lipids with their distinctive structures presents an opportunity for a reverse pathway analysis, namely to explore the basis of covalent binding and the link to the 12*R*-LOX pathway by working back from the KOH-released lipids to identify the structures and mechanism of synthesis of their covalently bound precursors.

## Experimental procedures

### Materials

Linoleic acid was purchased from Nu-Chek Prep. Cer-EOS of natural composition of long-chain fatty acid and sphingosine was extracted and purified from pig epidermis. Cer-EOS with C_30_ long-chain amide-linked fatty acid was a kind gift from Evonik. Cer-OS of natural long-chain fatty acid and sphingosine composition was prepared by alkaline hydrolysis of the fatty acids esterified in Cer-EOS. Cer-OS of C_30_ amide–linked long-chain fatty acid was a kind gift from Cayman Chemical. For TLC analysis, an authentic lipid mixture (standards purchased from Sigma–Aldrich) was prepared containing triglyceride, cholesterol ester, oleic acid, cholesterol, Cer-EOS (from Evonik), and phosphatidylcholine.

### Extraction of porcine and mouse epidermis

Sections of pig skin were collected within 1 h of animals being euthanized after unrelated experiments in the Department of Surgery and the tissue frozen at −80 °C. After thawing at room temperature for ∼1 h, the skin was placed in a 70 °C water bath for 60 s and the epidermis subsequently removed by scraping and peeling and either extracted or again stored at −80 °C. Free lipids were extracted from the epidermal tissue after cutting with scissors followed by homogenization in CHCl_3_/MeOH (2:1) using a Kinematica Polytron P10-35 operated up to full speed with a 20 cm diameter aggregate probe. As solids do not spin down well in CHCl_3_/MeOH (2:1), the proportions were adjusted to 1:1 for centrifugation and collection of the solvent. The homogenization was repeated four times, and then, the resuspended pellet was mixed overnight in CHCl_3_/MeOH (2:1) at 4 °C under argon. After a final collection of freely extractable lipids, the protein pellet was mixed with 1 M KOH in 95% MeOH and left overnight at room temperature under argon. After removal of solids by centrifugation, an equal volume of water was added to the alkaline solution and the pH neutralized with 1 N HCl and carefully brought down to pH 4 to 5. The solvent proportions were first adjusted to the Bligh and Dyer single-phase proportions of 1:1.25:2.5 (water:MeOH:CHCl_3_), and the phases were then split by addition of CHCl_3_ and water to give final proportions of 2:2.5:2.5, respectively (28). The solutions were centrifuged to cleanly separate the phases, the lower phase was collected, and the upper aqueous/MeOH phase was re-extracted with theoretical lower phase (prepared by mixing clean solvents in the Bligh and Dyer proportions). The combined lower phases were washed with a small volume of theoretical upper phase to remove any excess acid and then taken to dryness either by rotary evaporation or under a stream of nitrogen. Mouse epidermal extracts were prepared from C57BL/6 pups that had been euthanized in unrelated studies (approved Vanderbilt Institutional Animal Care and Use Committee protocol) and stored at −80 °C. Back and abdominal skin was removed and incubated in 2% dispase at 4 °C overnight, and the epidermis was peeled off and extracted on a smaller scale essentially as described previously.

### Preparation of human CEs

CEs were prepared by a modification of a previous method (32). Deidentified human breast skin was obtained from breast reduction surgery (Vanderbilt IRB protocol #100953 and also in compliance with the principles of the Declaration of Helsinki). The epidermis was isolated by scraping from the surface following incubation in 60 °C water for 1 min. Stratum corneum sheets were obtained by treatment of the epidermis with trypsin 0.05% (Gibco) solution for 30 min at 37 °C followed by vortexing and thoroughly rinsing off the living keratinocytes with distilled water. CEs were prepared from the stratum corneum sheets by boiling in an extraction buffer of 0.1 M Tris–HCl with 2% SDS, 5 mM EDTA, and 20 mM dithiothreitol for 10 min followed by centrifugation at 5000*g* for 15 min. The extraction and centrifugation were repeated a second time. The prepared CEs were fragmented by ultrasonication using a probe sonicator (Sonic Dismembrator Model 100; Fisher Scientific) at 30% power for 1 min on ice, in 5 s intervals with equal rest intervals between each sonication. The CE fragments were rinsed with extraction buffer twice and distilled water twice and then dried overnight in a lyophilizer. The dried CE fragments were treated with 1 M KOH in 95% ethanol/5% distilled water overnight at room temperature, neutralized with 1 M HCl, and extracted by the Bligh and Dyer method as described previously.

### Topical application of [^14^C]linoleic acid to whole mouse skin and *ex vivo* culture

Neonatal mouse pups (C57BL/6, 1–4 days old) were obtained within 1 h of being euthanized with CO_2_ followed by decapitation (approved Vanderbilt Institutional Animal Care and Use Committee protocol). Skin was isolated from the mouse pups and placed on a 4.1 cm^2^ culture insert (Thermo) dermis side down and cultured at air–liquid interface. CnT-Prime 3D barrier (CellnTec) was used as the culture medium. [^14^C]Linoleic acid (10^6^ CPM, 4.8 μg) dissolved in 35 μl 10% acetone in hexane was applied topically on the mouse skin (the solvent evaporated quickly) and incubated overnight at 37 °C in a 5% CO_2_ incubator. The skin was washed with PBS and treated with 1 mg/ml dispase II at 4 °C overnight. The epidermis was isolated from the skin, and epidermal lipids were extracted four times with CHCl_3_/MeOH and the free lipids analyzed by TLC as described later. The final protein pellet was mixed thoroughly with 1 M KOH in 95% MeOH and incubated overnight at room temperature and extracted as described previously.

The free ^14^C-labeled lipids from the *ex vivo* epidermis were analyzed in a TLC protocol designed to separate lipids of widely differing polarity and modified slightly from the literature (33). Lane 1 contained the ^14^C lipids, lane 2 unlabeled linoleic acid, and lane 3 a mixture of unlabeled authentic lipid standards. Samples were run on a silica gel HL channeled plate (5 × 20 cm; Analtech) using three successive solvent systems as follows: (i) CHCl_3_/MeOH/H_2_O/HAc (80:20:2:1) run successively to 1 cm (dried), and similarly to 2, 3, and 4 cm; (ii) CHCl_3_/MeOH/HAc (98:1.5:1) run to 10.5 cm; (iii) hexane/diethyl ether/HAc (90:10:1) run to 8.5 cm, dried then finally run to 16 cm. After development, lane 1 containing the ^14^C-labeled lipids was divided into 32 sections of 0.5 cm, scraped into scintillation vials, and counted in 3 ml Bio-Safe II cocktail. Then the unlabeled standards were detected by spraying with 3% CuSO4/8% phosphoric acid (dissolved in 90 ml water) and heating the plate for 10 min at 140 °C. An image of the plate was then taken for incorporation into the figure.

The protein-bound ^14^C lipids from base hydrolysis of the protein pellet were run on both RP-HPLC and SP-HPLC before counting the radioactive HPLC fractions across the peaks of the polar or less polar lipids. RP-HPLC-UV used a Waters Symmetry C18 column (5 μ, 25 × 0.46 cm) run at a flow rate of 1 ml/min with CH_3_CN/H_2_O/HAc (50:50:0.01) for 15 min, switching to the proportions 80:20:0.01 for 15 to 30 min. Fractions were counted (0.25 ex 1 ml), and the radiolabeled peak of interest was run again by SP-HPLC-UV with a solvent of hexane/isopropanol/HAc (90/10/0.02 by volume), at a flow rate of 0.5 ml/min, with collection of fractions every 30 s for liquid scintillation counting.

### HPLC-UV analyses

Aliquots of the extracts of covalently bound lipids were analyzed by SP-HPLC using a Thomson Advantage 5 μm silica column (25 × 0.46 cm), with a solvent of hexane/isopropanol/HAc (90/10/0.02 by volume), typically at a flow rate of 1 ml/min, with on-line UV detection at 205, 220, 235, and 270 nm (Agilent 1100 series diode array detector). Further purification of the polar and less polar lipids was achieved by RP-HPLC using a Waters 5 μm Symmetry column (25 × 0.46 cm) using a solvent of acetonitrile/water/HAc (70/30/0.01, by volume) run at 1 ml/min.

### LC–MS analyses

High-resolution LC–MS of the polar and less polar lipids used a Thermo Q Exactive HF—HF Hybrid Quadrapole-Orbitrap (Thermo Fisher Scientific). RP-HPLC analysis was performed with electrospray ionization in the positive and negative ion modes. An Agilent C18 5 μ column (15 × 0.46 cm) was eluted isocratically with acetonitrile/water/HAc (70:30 by volume, 10 mM in ammonium acetate) at a flow rate of 0.5 ml/min. The electrospray voltage was set at 5.0 kV; sheath and auxiliary gas pressure at 40.00 and 10.00 ψ, respectively; capillary temperature at 320 °C. Scan range is from 100 to 1000 *m/z* and in-source collision-induced dissociation 10.0 ev.

### Derivatization and GC–MS

To prepare methyl ester derivatives and circumvent the adduction of diazomethane to conjugated enones (*e.g.*, (34)), samples were dissolved in 5 or 10 μl MeOH in a 1 ml Reacti-Vial and placed on ice; two to three drops of ethereal diazomethane were added, immediately mixed, and within 10 s, the sample was placed under a stream of nitrogen for rapid removal of the excess reagent. Methoxime derivatives were prepared by reaction with 10 μl of 10 mg/ml methoxylamine hydrochloride in pyridine; the typical overnight and room temperature reaction conditions gave only minor transformation of the lipids studied here, and overnight, at room temperature plus 1 h at 60 °C was required to give >50% conversion to the methoxime derivative. Catalytic hydrogenation was accomplished using ∼1 mg palladium on carbon in 100 μl EtOH and bubbling with H_2_ for 2 min followed by addition of EtOAc and water, thorough mixing, and recovery of the organic phase. TMS ether or ester derivatives were prepared by treatment with 10 μl BSTFA plus 2 μl pyridine for at least 1 h at room temperature. Reductions with NaBH_4_ were carried out in 100 μl EtOH with addition of sufficient NaBH_4_ to see traces of solid remaining and reaction overnight at room temperature. After addition of 100 μl 1 M KH_2_PO_4_ plus 200 μl water, and extraction with dichloromethane or EtOAc and a final wash of the solvent with water, subsequent analysis as the TMS ether derivatives by GC–MS showed three products in the relative ratio of 1:1:3 and eluting in the order methyl ester TMS ether, diTMS ether (the C_1_ carboxyl having been reduced to alcohol), and (by transesterification in the alkaline EtOH), ethyl ester TMS ether. Because of the limited amounts of analytes available, some reductions with NaBH_4_ were carried out on the remaining MeTMS derivatives following analysis by GC–MS, which, after another treatment with BSTFA, gave results as just described.

Aliquots of ∼10 to 100 ng were analyzed by GC–MS utilizing a DB-5 column (30 m × 0.25 mm; Agilent) on a Thermo-Finnigan DSQ mass spectrometer operated in the positive ion EI mode (70 eV) with temperature programming at 10 °C/min from 150 to 300 °C.

### NMR analyses

^1^H NMR and ^1^H,^1^H COSY, HSQC, and HMBC NMR experiments were acquired using a 14.0 T Bruker magnet equipped with a Bruker AV-III console operating at 600.13 MHz. The 2D NOESY data for the polar lipid sample in d_6_-benzene, along with COSY, HSQC, and HMBC spectra, were acquired using an 18.8 T Bruker magnet equipped with a Bruker AV-III console operating at 799.75 MHz. All spectra were acquired in 3 mm NMR tubes using a Bruker 5 mm TCI cryogenically cooled NMR probe. Chemical shifts were referenced internally to d_6_-benzene (7.16 ppm), and in some experiments to CDCl_3_ (7.26 ppm).

## Data availability

Data are contained within the main article and in the supporting information. For any queries regarding the article data, please contact the corresponding author.

## Supporting information

This article contains supporting information (Figs. S1–S11).

## Conflict of interest

The authors declare that they have no conflicts of interest with the contents of this article.
