# Supplementary material for: Two C18 hydroxy-cyclohexenone fatty acids from mammalian epidermis: Potential relation to 12R-lipoxygenase and covalent binding of ceramides
Source: J Biol Chem. 2023 Apr 21;299(6):104739. doi: 10.1016/j.jbc.2023.104739 (PMC10209020; doi:10.1016/j.jbc.2023.104739)

Supporting Information

Two C18 hydroxy-cyclohexenone fatty acids from mammalian epidermis: Potential relation to 12*R*-lipoxygenase and covalent binding of ceramides

Alan R. Brash, Saori Noguchi, William E. Boeglin, M. Wade Calcutt, Donald F. Stec, Claus Schneider, and Jason M. Meyer

Figure S1: SP-HPLC analysis of lipids covalently bound to epidermal protein

Figure S2: Full 1D spectrum and COSY analysis of the polar lipid methyl ester, 800 MHz in d_6-benzene

Figure S3: HSQC analysis of the polar lipid methyl ester, 800 MHz in d_6-benzene

Figure S4: Partial 1D spectrum (0.8 – 2.8 ppm) and COSY analysis of the polar lipid methyl ester, 600 MHz in $CDCl_3$

Figure S5: HMBC analysis of the polar lipid methyl ester, 600 MHz in $CDCl_3$

Figure S6: Full NOESY spectrum of the polar lipid methyl ester, 800 MHz in d_6-benzene

Figure S7: Detail view of the NOESY spectrum (0.8 – 2.6 ppm) of the polar lipid methyl ester, 800 MHz in d_6-benzene

Figure S8: Full 1D spectrum and COSY analysis of the less polar lipid methyl ester, 600 MHz in d_6-benzene

Figure S9: Detailed view of the HMBC spectrum (0.8 – 2.6 ppm) of the less polar lipid methyl ester, 600 MHz in d_6-benzene

Figure S10: TLC ^{14}C-profile of freely-extractable lipids from epidermis of two additional whole mouse skins incubated overnight with [^{14}C]linoleic acid.

Figure S11: RP-HPLC (235 nm and 205 nm profiles) of the less polar lipid recovered from covalently-bound lipids of whole mouse skin incubated with [^{14}C]linoleic acid *ex vivo*.

Figure S1
SP-HPLC analysis of lipids covalently bound to epidermal protein
Analysis of the covalently-bound lipids used the same isocratic solvent of hexane/IPA/glacial acetic acid (90:10:0.02 by volume) although with differing silica columns or flow rates. A: porcine epidermal lipids analyzed using a Thomson Advantage 5 µm silica column (25 x 0.46 cm) and a flow rate of 0.5 ml/min. B: mouse pup epidermal lipids analyzed using an Alltima 3 µm silica column (10 x 0.21 cm) and a flow rate of 0.5 ml/min. C: washed human epidermal corneocyte envelopes analyzed using the same column as in panel A with a flow rate of 1 ml/min. The recordings shown are 205 nm (blue) and 235 nm (red).

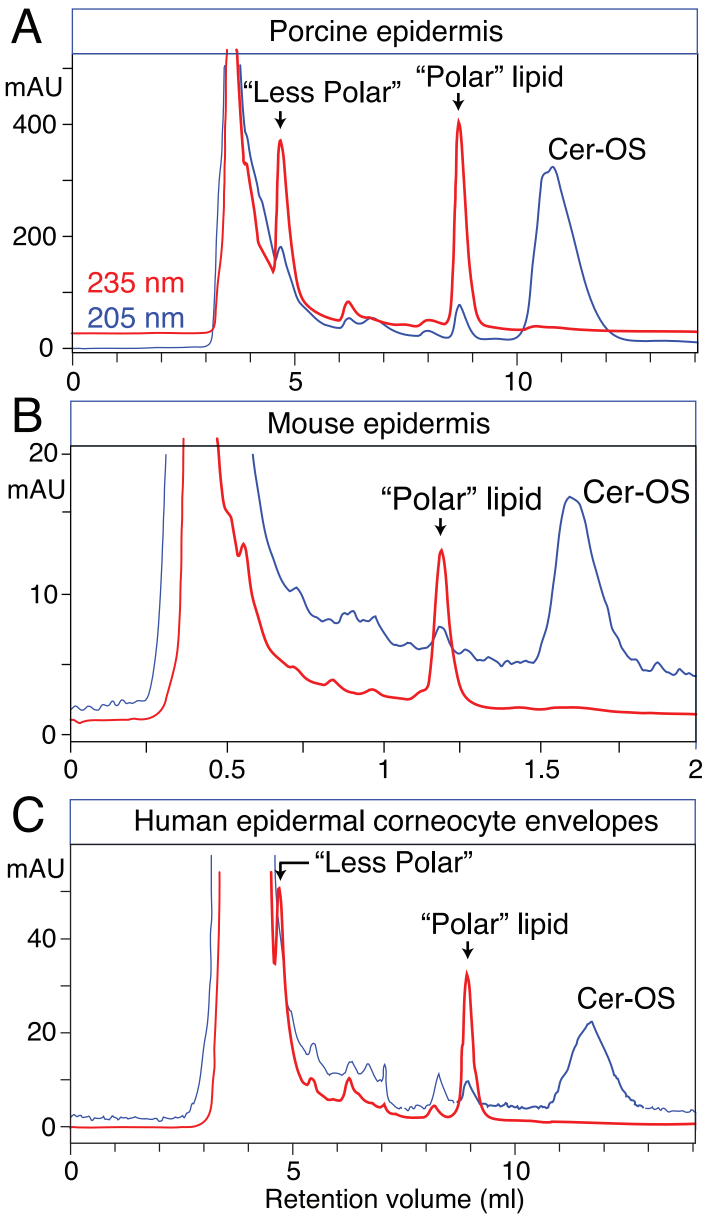

Figure S2
Full 1D spectrum and COSY analysis of the polar lipid methyl ester, 800 MHz in d_6-benzene

The full spectrum for the polar lipid methyl ester, ^{1}H-NMR Chemical shift δ, multiplicity, number of protons, proton number, and *J*: (800 MHz, C_6D_6) δ (ppm) 3.83 (m, 1, H10), 3.35 (s, 3, OC<u>H</u>$_3$), 2.49 (m, 1, H15b, 2.45 (ddd, 1, H12b, *J* = 4.5, 7.5, 16.5 Hz), 2.41, (m, 1, H15a), 2.325 (ddd, 1, H8b, *J* = 5.24, 10.9, ~12 Hz), 2.18 (ddd, 1, H8a, *J* = 5.8, 10.75, 12.5 Hz), 2.11 (t, 2, H2, $J_{2,3}$ = 7.4 Hz), 2.025 (ddd, 1, H12a, *J* = 4.8, 9.8, 16.5 Hz), 1.61 (m, 1, H11b), 1.545 (m, 2, H3), 1.47 (m, 3, H11a, H16), 1.38, m, H7b, H17), 1.33 (m. 1, H7a), 1.21 (m, 2, H6) , 1.18 (m, 4, H4, H5), 0.93 (t, 3, H18), 0.83 (d, 1 (area of ~0.5), -O<u>H</u>).

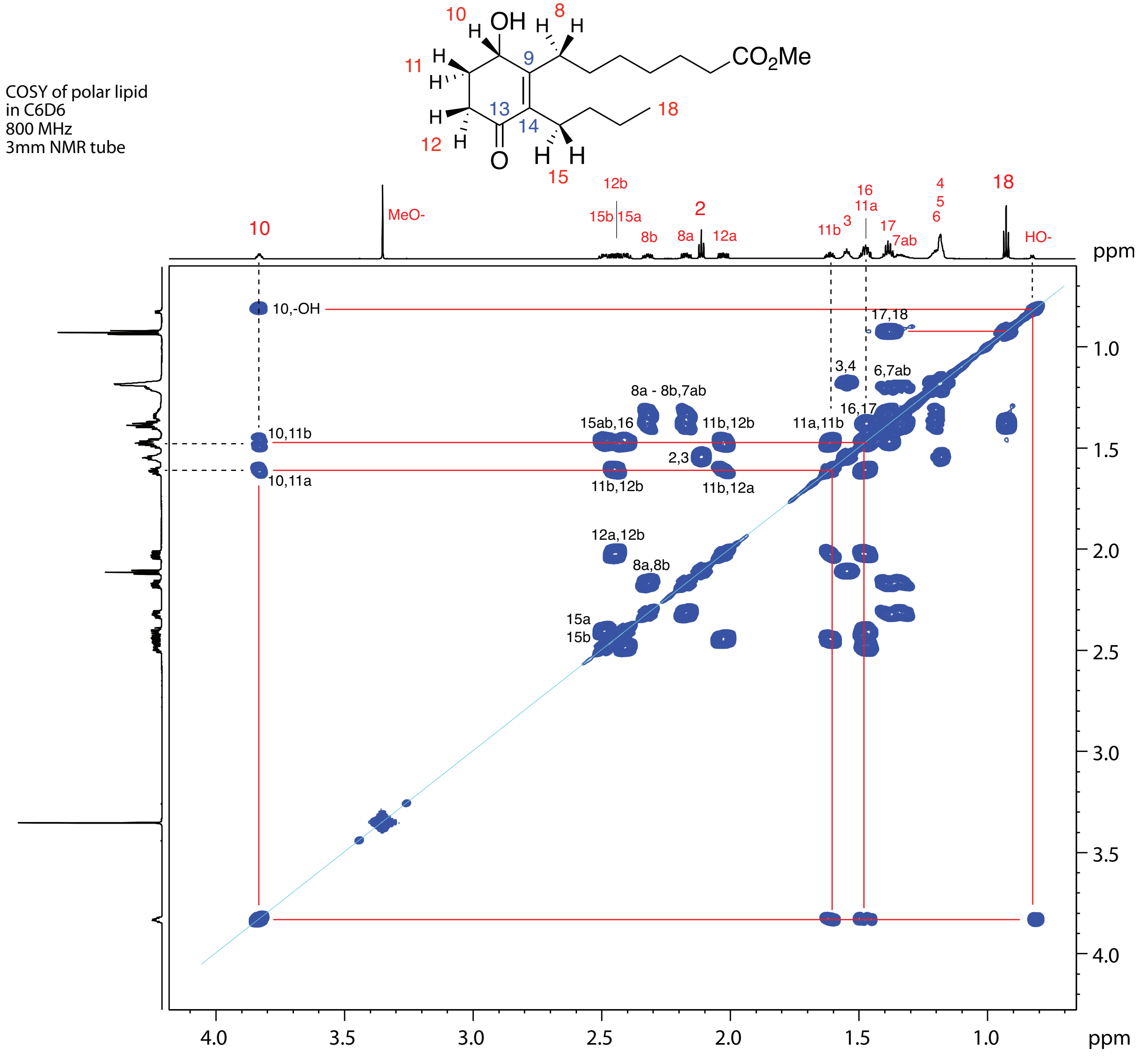

Figure S3
HSQC analysis of the polar lipid methyl ester, 800 MHz in d_6-benzene

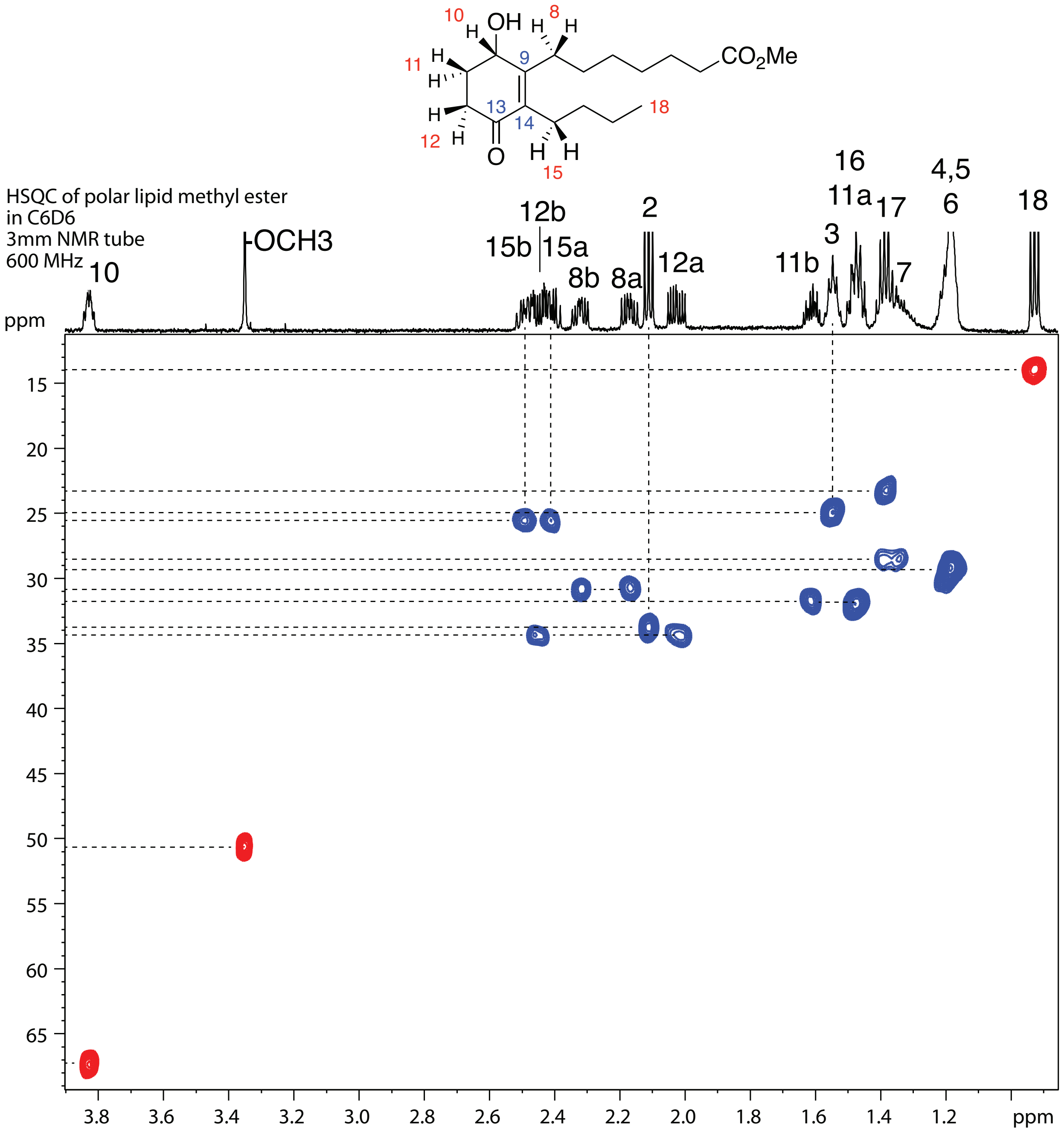

Figure S4

Partial 1D spectrum (0.8 – 2.8 ppm) and COSY analysis of the polar lipid methyl ester, 600 MHz in $CDCl_3$

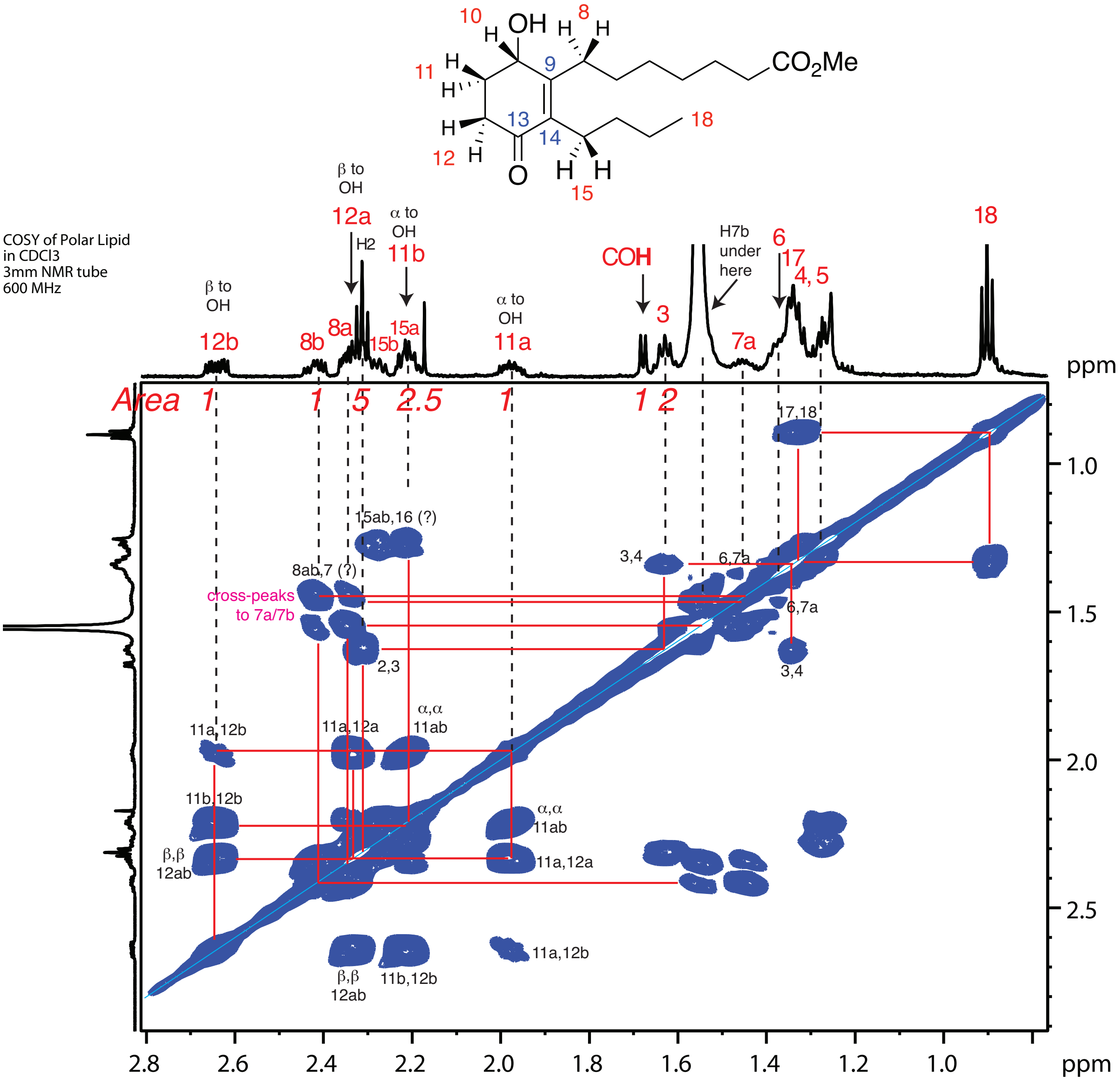

Figure S5
HMBC analysis of the polar lipid methyl ester, 600 MHz in $CDCl_3$

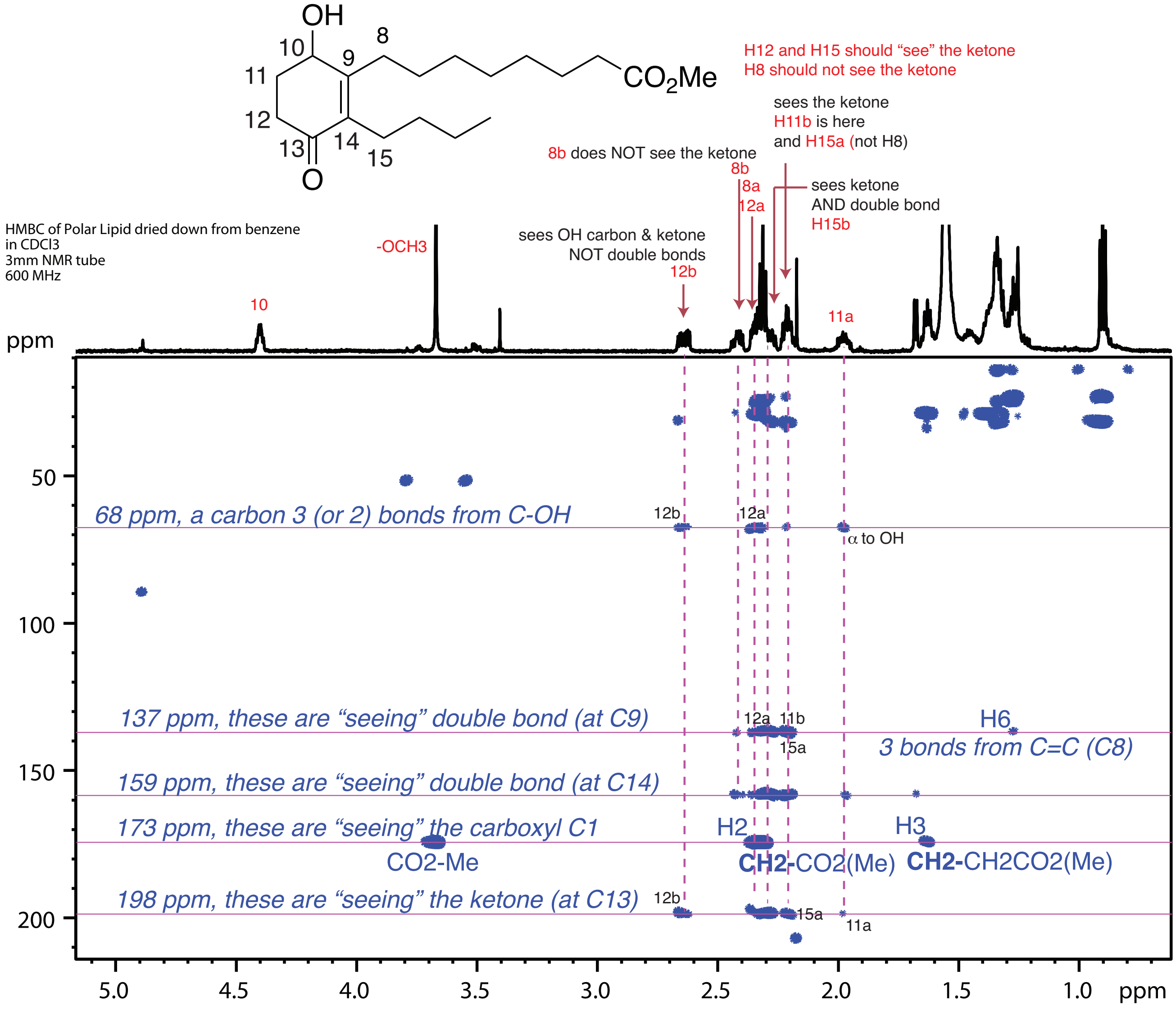

Figure S6
Full NOESY spectrum of the polar lipid methyl ester, 800 MHz in d_6-benzene

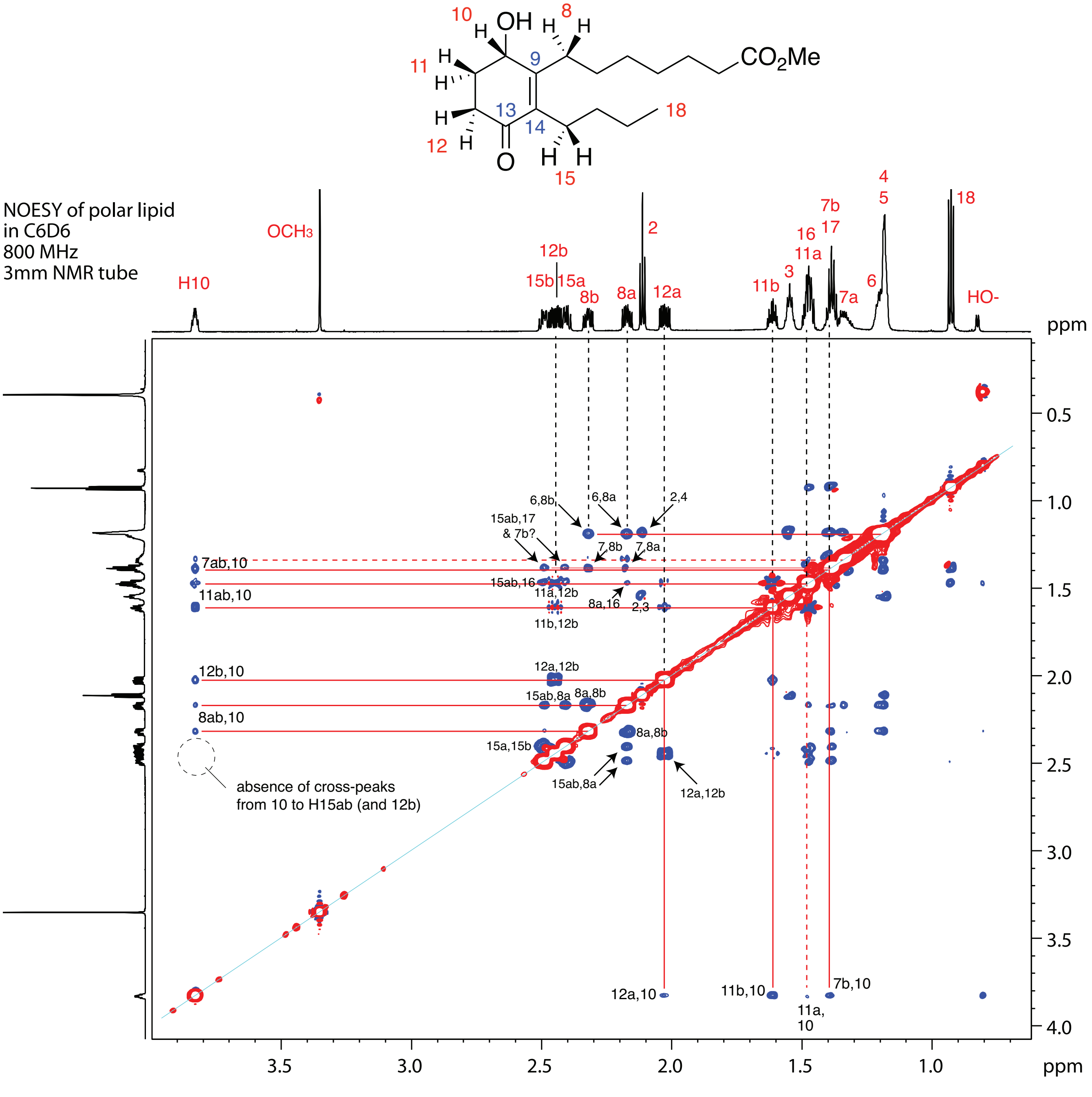

Figure S7
Detail view of the NOESY spectrum (0.8 – 2.6 ppm) of the polar lipid methyl ester, 800 MHz in d_6-benzene

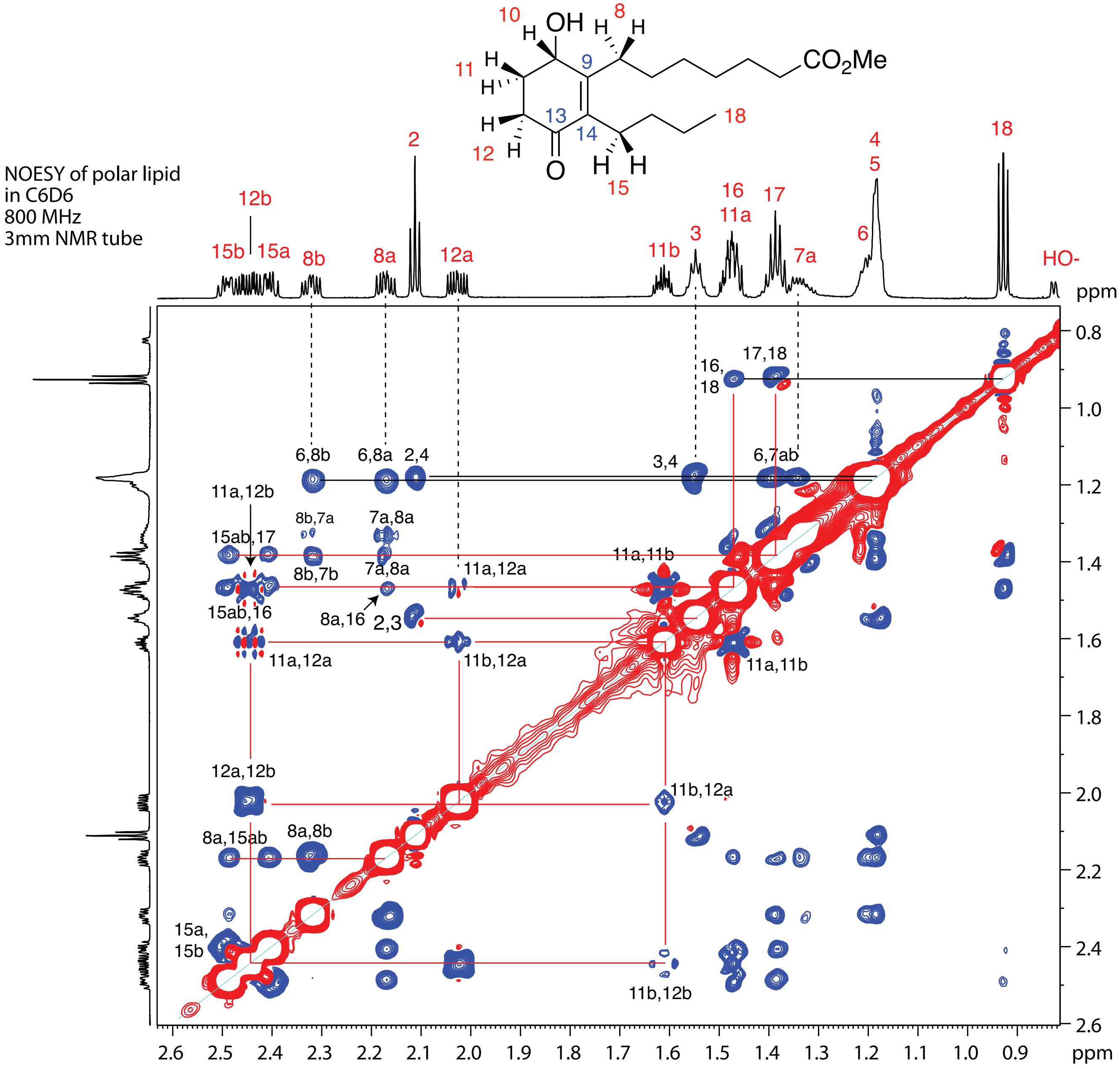

Figure S8
Full 1D spectrum and COSY analysis of the less polar lipid methyl ester, 600 MHz in d_6-benzene

Less polar methyl ester, ^{1}H-NMR Chemical shift δ, multiplicity, number of protons, proton number, and *J*: (600 MHz, C_6D_6) δ (ppm) 4.11 (d, 1, O<u>H</u>, J = 1.7 Hz), 3.85 (ddd, 1, H10, J = 1.7, 5.5, 13.6 Hz), 3.33 (s, 3, OC<u>H</u>$_3$), 2.47 (m, 1, H7b), 2.22 (m, 1, H7a), 2.08 (t/m, 3, H2, H11b), 1.84 (m, 2, , 1.82 (m, 1, H11b), 1.71 (1, apparent br d, H12b), 1.59 (ddd, 1, H12a), 1.55 (m, 2, H3), 1.36 (m, 2, H6), 1.25-1.17 (m, 6, H4, H5, H17), 1.16 – 1.05 (m, 4, H15,H16), 0.86 (t, 3, H18).

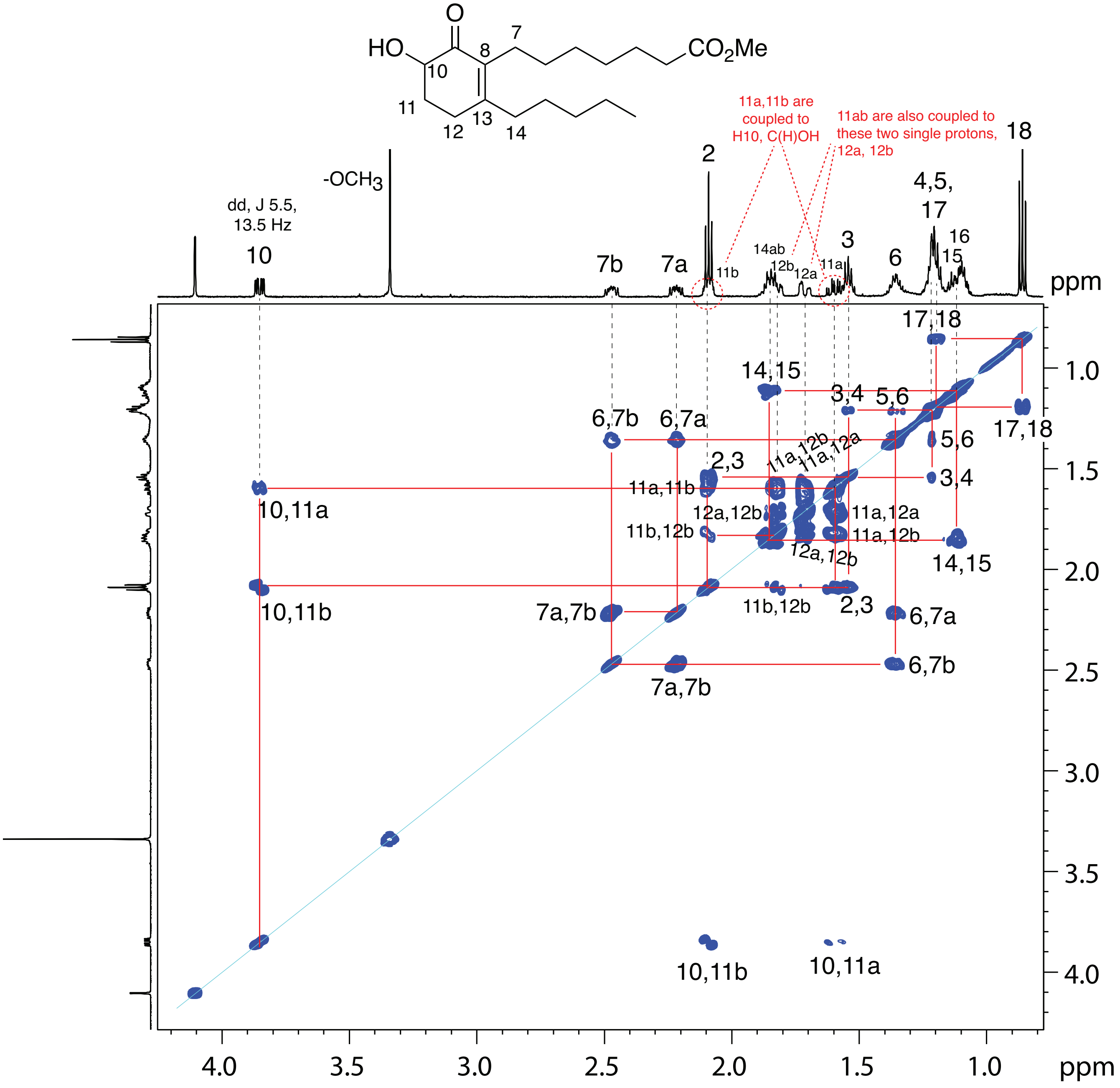

Figure S9
Detailed view of the HMBC spectrum (0.8 – 2.6 ppm) of the less polar lipid methyl ester, 600 MHz in d_6-benzene

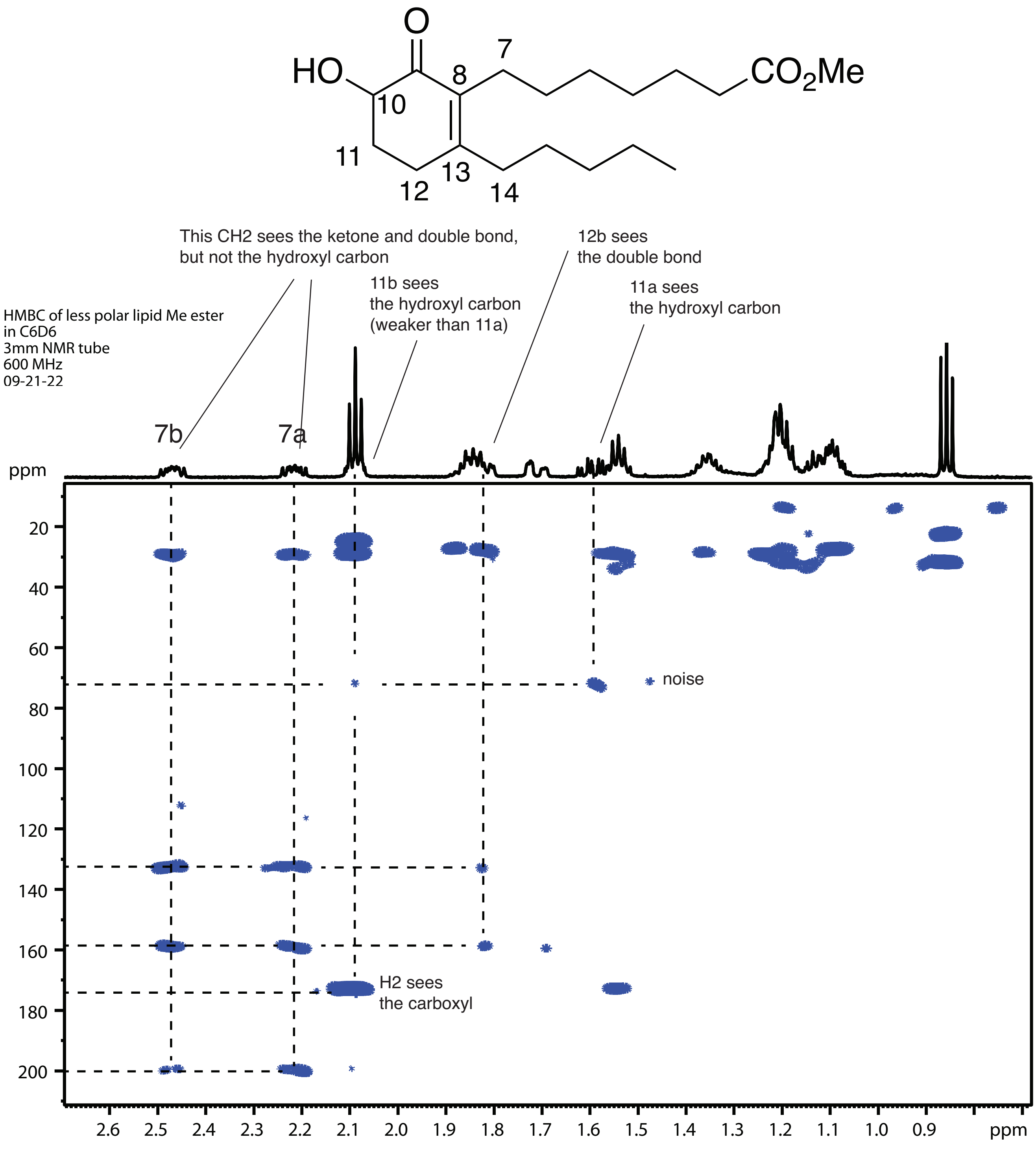

Figure S10
TLC ^{14}C-profile of freely-extractable lipids from epidermis of two additional whole mouse skins incubated overnight with [^{14}C]linoleic acid.
Abbreviations: Chol., cholesterol; TG, triglyceride; LA linoleic acid; Cer, ceramide; PL, phospholipid

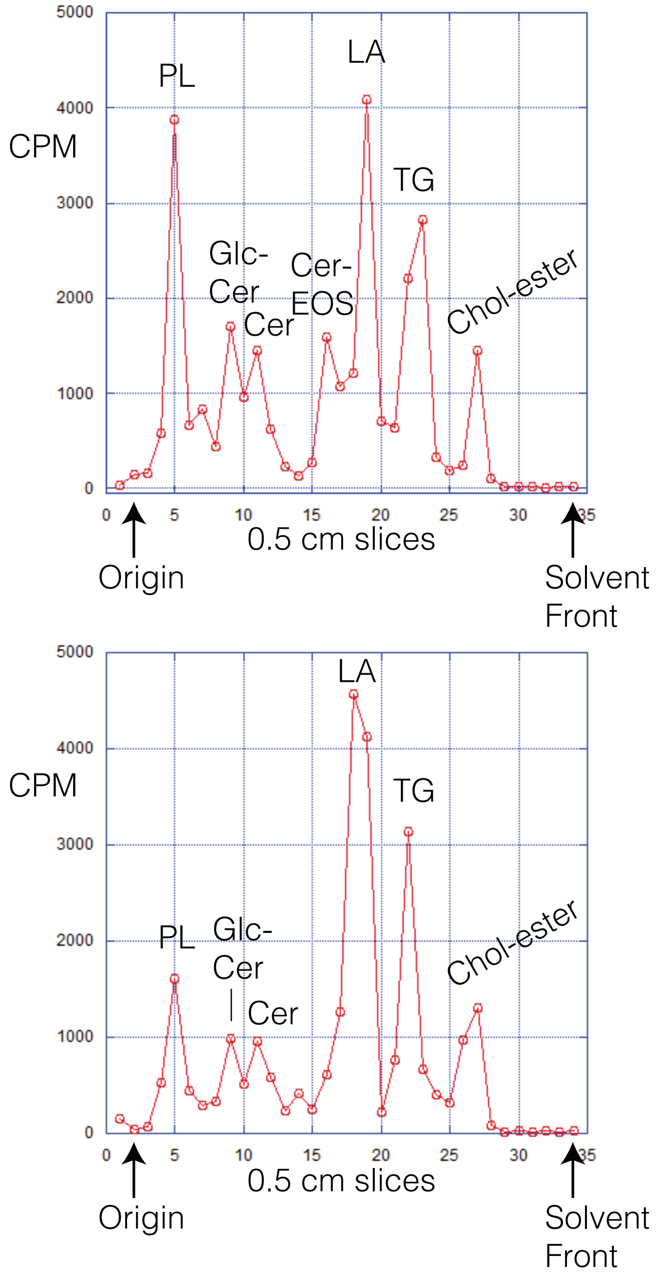

Figure S11

RP-HPLC (235 nm and 205 nm profiles) of the less polar lipid recovered from covalently-bound lipids of whole mouse skin incubated with [^{14}C]linoleic acid *ex vivo*. The less polar lipid was first isolated by SP-HPLC then further purified as shown here by RP-HPLC.

Column: Waters Symmetry 5 µm C18 (15 x 0.2 cm); solvent CH_3CN/H_2O/glacial acetic acid (50:50:0.01 by volume); flow rate 0.3 ml/min; fractions collected every 0.5 min and counted for at least 20 min each.

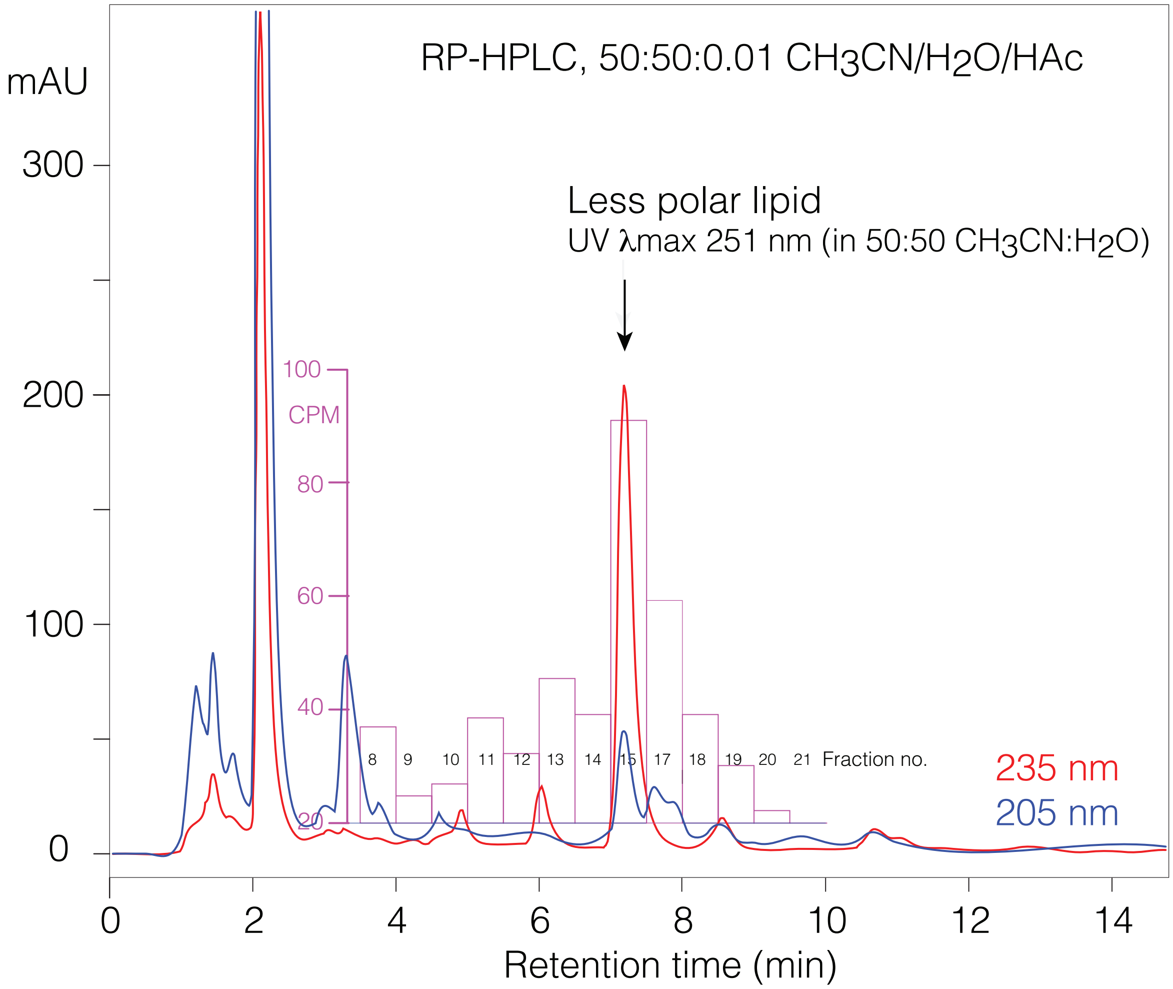

Supplement: Supporting information [file mmc1.pdf]
